# RhoE is required for contact inhibition and negatively regulates tumor initiation and progression

**DOI:** 10.18632/oncotarget.4127

**Published:** 2015-05-12

**Authors:** Marta Hernández-Sánchez, Enric Poch, Rosa M. Guasch, Joaquín Ortega, Inmaculada López-Almela, Ignacio Palmero, Ignacio Pérez-Roger

**Affiliations:** ^1^ Universidad CEU-Cardenal Herrera, Facultad de Ciencias de la Salud, Dep Ciencias Biomédicas, Moncada, Spain; ^2^ Centro de Investigación Príncipe Felipe, Rho Signaling in Neuropathologies, Valencia, Spain; ^3^ Universidad CEU-Cardenal Herrera, Facultad de Veterinaria, Dep. PASACTA, Moncada, Spain; ^4^ Instituto de Investigaciones Biomédicas “Alberto Sols” CSIC-UAM, Madrid, Spain; ^5^ Departament de Biologia cellular, Fisiologia i Immunologia, Universitat Autònoma de Barcelona, Cerdanyola del Vallés, Spain

**Keywords:** contact inhibition, metastasis, RhoE, tumor suppression, p27^Kip1^

## Abstract

RhoE is a small GTPase involved in the regulation of actin cytoskeleton dynamics, cell cycle and apoptosis. The role of RhoE in cancer is currently controversial, with reports of both oncogenic and tumor-suppressive functions for RhoE. Using RhoE-deficient mice, we show here that the absence of RhoE blunts contact-inhibition of growth by inhibiting p27^Kip1^ nuclear translocation and cooperates in oncogenic transformation of mouse primary fibroblasts. Heterozygous RhoE*^+/gt^* mice are more susceptible to chemically induced skin tumors and RhoE knock-down results in increased metastatic potential of cancer cells. These results indicate that RhoE plays a role in suppressing tumor initiation and progression.

## INTRODUCTION

RhoE/Rnd3 is an atypical member of the Rho family of proteins that negatively regulates the RhoA-ROCK pathway [1-5]. RhoE overexpression inhibits cellular proliferation, blocking the cell cycle in G1 [6-9]. Besides, RhoE is regulated along the cell cycle, accumulating in G1 and being rapidly degraded at the G1/S transition in a proteasome-dependent manner, and it also accumulates in primary fibroblasts reaching confluency [9]. RhoE is also induced by genotoxic stress in a p53 dependent fashion, acting as a pro-survival factor [10, 11].

The role of RhoE in cancer is not clear at present. Some reports suggest a possible tumor suppressor role for RhoE in human cancer and metastasis [7, 12-19]. However, additional evidence suggests a positive correlation between RhoE expression and malignancy [20-26].

By using mice in which RhoE expression has been ablated by a gene-trap cassette [27], we show here that this protein is dispensable for normal cellular proliferation, but its absence: a) causes lack of contact inhibition; b) cooperates with oncogenes in cellular transformation; c) increases susceptibility to chemical carcinogens *in vivo*; and d) increases the metastatic potential of cancer cells. These results demonstrate that RhoE contributes to tumor suppression.

## RESULTS

### RhoE is necessary for contact inhibition

**Figure 1:**
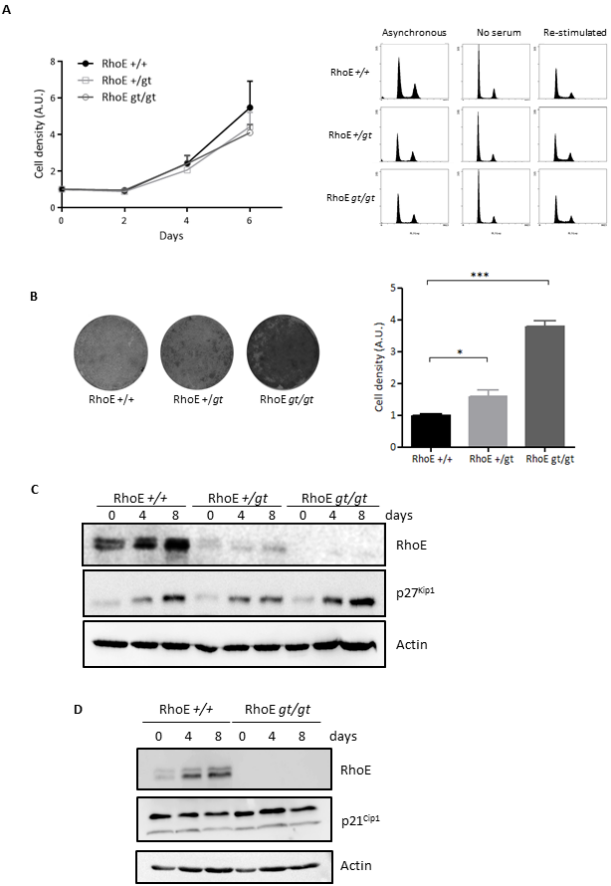
RhoE is a mediator of contact inhibition **A.** Lack of RhoE expression does not affect cell proliferation. Left: Primary MEFs were grown in DMEM-10% FBS and fixed at the indicated time points. Cell density was measured by crystal violet staining. Data (referred to time 0) from three independent experiments are shown as Mean+SEM. A.U.: arbitrary units. Right: Cell cycle profile of primary MEFs growing in 10% FBS (Asynchronous), serum-starved for 48 h (No serum) or re-stimulated for 16 h after serum-starvation (Re-stimulated) was analyzed by flow cytometry after DNA staining with Propidium Iodide. **B.** RhoE deficient cells are not contact inhibited. RhoE^+/+^, RhoE^+/*gt*^ and RhoE^*gt/gt*^ primary MEFs were kept in culture for 15 days and cell density was measured by crystal violet staining. Pictures show examples of the plates after staining. The graph shows the Mean+SEM of three independent experiments (*p < 0.05 and ***p < 0.001 in a Student's *t* test). A.U.: arbitrary units. **C.** p27^Kip1^ accumulates normally in high density cultures in the absence of RhoE. Primary MEFs as in B were kept in culture for 8 days with medium-change every 48 h. At the indicated time-points, the expression of RhoE and p27^Kip1^ was analyzed by Western blotting. Actin was used as a loading control. **D.** p21^Cip1^ expression does not change in the absence of RhoE expression. RhoE^*+/+*^ and RhoE^*gt/gt*^ primary MEFs were kept in culture for 8 days and the expression of p21^Cip1^ and RhoE was analyzed as in **C.**

In order to test the role of RhoE in the control of cell proliferation, we analyzed the growth of primary Mouse Embryo Fibroblasts (MEFs) from RhoE deficient mice (RhoE*^gt/gt^*) as well as from wild-type (RhoE*^+/+^*) and hemizygous (RhoE*^+/gt^*) animals. MEFs of the three genotypes showed similar growth rates (Figure 1A, left) and undistinguishable cell cycle profiles either in asynchronous growth, during serum starvation or upon serum re-addition (Figure 1A, right panels). From these results, the absence of RhoE expression does not have a clear effect on normal cell proliferation.

RhoE accumulates in cells growing at high density [9], suggesting that it might be involved in the regulation of contact inhibition. To test this hypothesis, we measured the cell density reached by primary fibroblasts from RhoE*^gt/gt^* and RhoE*^+/gt^* mouse embryos kept in culture for 15 days and compared it to that of RhoE*^+/+^* cells. Figure 1B shows that RhoE*^gt/gt^* MEFs reached densities four times higher than wild type cells. Interestingly, RhoE*^+/gt^* MEFs displayed an intermediate behavior, suggesting a possible gene dosage effect.

The cell cycle inhibitor p27^Kip1^ mediates contact inhibition in response to cadherins [28-30]. Also, RhoE accumulates in high density cultures and follows the same expression pattern as p27^Kip1^ [9]. Therefore, we reasoned that there could be a functional link between these two proteins. To test whether RhoE could affect the expression of p27^Kip1^, we analyzed the levels of this protein at different time points along the experiment (Figure 1C). In wild type MEFs, both p27^Kip1^ and RhoE accumulated as they reached saturation. p27^Kip1^ also accumulated in RhoE*^+/gt^* and in RhoE*^gt/gt^* MEFs to the same extent as in wild type cells, indicating that, in the absence of RhoE expression, primary MEFs are able to keep proliferating to high density even in the presence of high p27^Kip1^ levels (Figure 1C). We also analyzed the expression along the experiment of other cell cycle regulators, such as the CDK inhibitor p21^Cip1^ or cyclins D and E, but there was no difference between wild type and RhoE*^gt/gt^* MEFs (Figure 1D and data not shown).

### RhoE is required for correct localization of p27^Kip1^ to the nucleus in high density cultures

**Figure 2:**
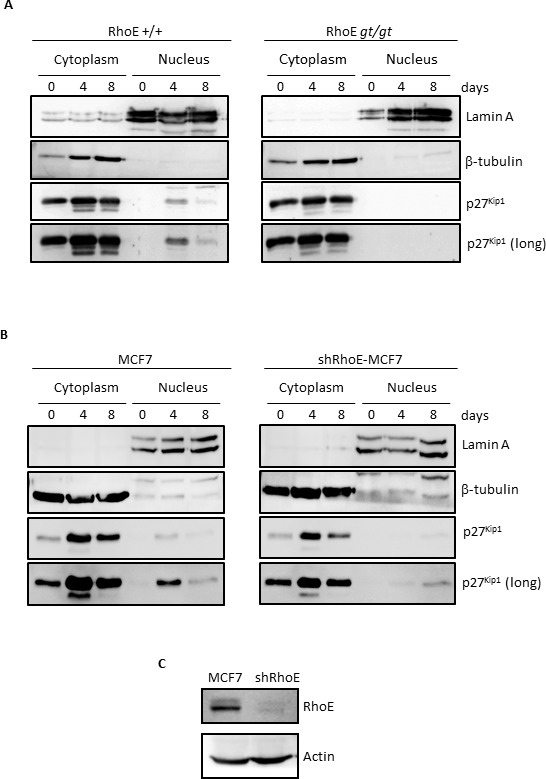
RhoE is necessary for nuclear localization of p27^Kip1^ in high density cultures **A.** RhoE^+/+^ and RhoE^*gt/gt*^ primary MEFs were kept in culture for 8 days and at the indicated time points nuclear-cytoplasm fractionation was performed as indicated in the Methods section. The expression of p27^Kip1^ in both fractions was analyzed by Western blotting. Lamin A and β-tubulin were used as controls for purity of the nuclear and cytoplasmic fractions, respectively. The bottom panel shows a longer exposure of the p27^Kip1^ blot. **B.** The expression of p27^Kip1^ in the nuclear and cytoplasmic fractions of MCF7 and shRhoE-MCF7 cells in high density cultures was analyzed as in **A. C.** RhoE silencing in MCF7 cells was analyzed by Western blotting in extracts from control or shRhoE-transduced MCF7 cells.

Subcellular localization is crucial for p27^Kip1^ function. Although p27^Kip1^ works as an inhibitor of cell cycle progression when located in the nucleus, it shows oncogenic properties as a cytoplasmic protein [31, 32]. We therefore analyzed the localization of p27^Kip1^ in MEFs reaching high densities. As shown in Figure 2A, p27^Kip1^ entered the nucleus in RhoE*^+/+^* cells after 4 days in culture (left panel). In contrast, p27^Kip1^ could not be detected in the nuclear fraction of cells lacking RhoE expression and remained in the cytoplasmic fraction throughout the length of the experiment (right panel).

We wanted to extend this finding to other cell types. For that purpose we used the MCF7 human breast adenocarcinoma cell line, in which we knocked-down RhoE expression by using shRNA (Figure 2C). After 4 days in culture, p27^Kip1^ accumulated in MCF7 cells and was also abundant in the nuclear fraction (Figure 2B, left panel). In contrast, p27^Kip1^ nuclear accumulation was dramatically blunted when RhoE expression was knocked-down and p27^Kip1^ could only be detected in the nuclear fraction after 8 days in culture and in longer exposed films (Figure 2B, right panel).

### Lack of RhoE expression facilitates spontaneous immortalization and oncogenic transformation

The ability of RhoE*^gt/gt^* primary MEFs to reach higher densities in culture could reflect suppression of senescence. To test this hypothesis, we performed a 3T3 serial passage protocol [33] to follow entry into, and exit from, senescence. Our results revealed no significant differences in the passage number at which cells entered senescence (RhoE*^+/+^*, 6.0±1.3; RhoE*^+/gt^*, 5.1±0.6; RhoE*^gt/gt^*, 5.6±2.7). However, while RhoE*^+/+^* cells immortalized at passage number 16.0±1.5, RhoE*^+/gt^* and RhoE*^gt/gt^* MEFs exited senescence at an earlier passage (9.8±0.9 and 10.3±0.8, respectively; *p* < 0.05 vs RhoE*^+/+^* in a Student's *t* test).

**Figure 3:**
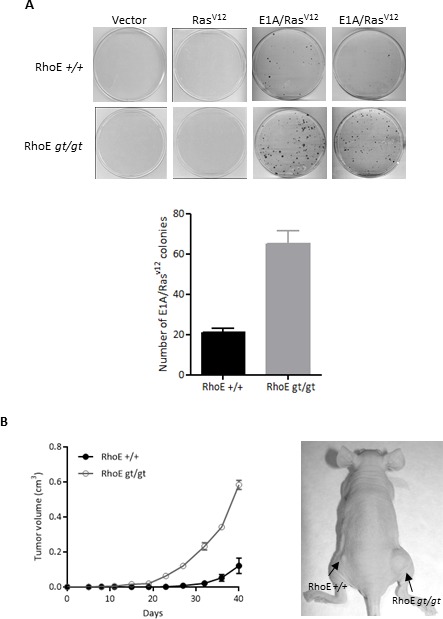
RhoE deficiency cooperates with E1A and Ras^V12^ **A.** Primary MEFs were infected with retroviruses containing empty pLPC (Vector), pLPC-Ras^V12^ or pLPC-E1A/Ras^V12^, selected for 3 days with Puromycin and kept in culture for 1 week (Ras^V12^ or E1A/Ras^V12^) or 3 weeks (empty vector). Transformed colonies were stained with crystal violet and counted. Mean+SD of two independent experiments is plotted (bottom graph). **B.** RhoE^+/+^ and RhoE^*gt/gt*^ MEFs previously infected with pLPC-E1A/Ras^V12^ were subcutaneously injected into the left and right flanks of nude mice (4×10^5^ cells in 100 μl of PBS per injection). Tumor volume was calculated every 4 days. Mean±SEM is plotted (left) and a representative example is shown (right). Differences between RhoE^+/+^ and RhoE^*gt/gt*^ are significant in a 2 way ANOVA (*p* < 0.0001).

We next asked whether the absence of RhoE expression would also increase the transforming ability of oncogenes in a colony formation assay. Oncogenic Ras^V12^ alone was unable to induce colony formation in wild type or RhoE*^gt/gt^* primary MEFs, while the combination E1A/Ras^V12^ resulted in the apparition of colonies. However, RhoE*^gt/gt^* MEFs showed a threefold increase in the number of colonies compared to RhoE*^+/+^*, indicating that the absence of RhoE cooperates with E1A/Ras^V12^ in oncogenic transformation (Figure 3A). Finally, we tested the ability of the transformed cells to form tumors in nude mice. Tumors derived from E1A/Ras^V12^-transformed RhoE*^gt/gt^* MEFs grew more rapidly than those expressing RhoE (Figure 3B). This indicates that the absence of RhoE expression facilitates transformation and tumorigenesis.

### Absence of RhoE expression increases susceptibility to chemically induced skin tumors

**Figure 4:**
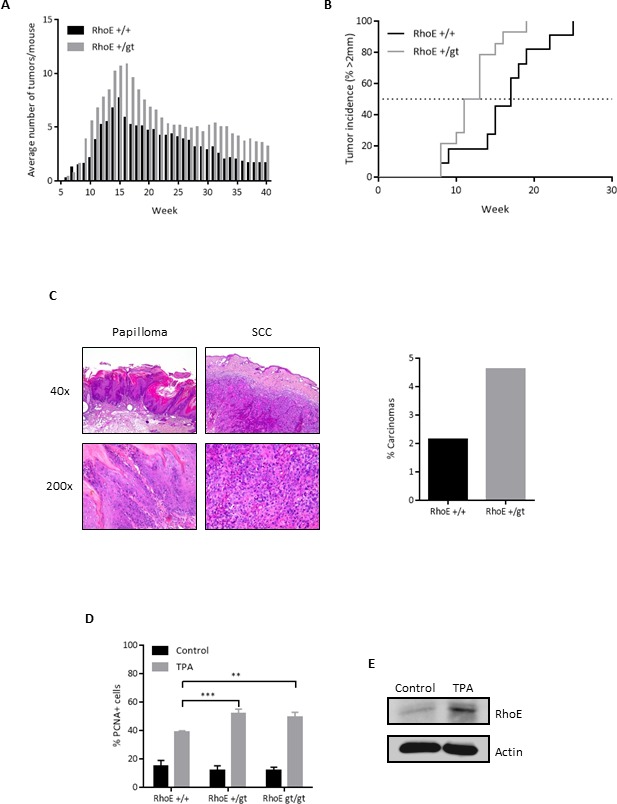
RhoE protects from DMBA/TPA-induced skin tumors in mice **A.** Average number of tumors in RhoE^+/+^ and RhoE^+/*gt*^ mice (n = 14 each) treated with DMBA/TPA. TPA treatment was discontinued after week 15. Differences between both genotypes were significant in a 2 way ANOVA (*p* < 0.0001). **B.** Percentage of mice having tumors bigger than 2 mm. *p* = 0.0145 in a Mantel-Cox test. **C.** After the DMBA/TPA treatment, mice were sacrificed and lesions classified as papilloma or squamous cell carcinoma (SCC). Representative images of papilloma (left) and SCC (right) from a RhoE^+/*gt*^ mouse are shown. Percentage of carcinomas relative to the maximum number of papillomas is represented (right). In RhoE^+/+^ mice, 1 carcinoma was found from a maximum of 47 papillomas at week 15, whereas in the case of RhoE^+/*gt*^ mice, 7 carcinomas from 151 papillomas were found. Thus, the conversion rate from papilloma to carcinoma was 2.1% for RhoE^+/+^ and 4.6% for RhoE^+/*gt*^ mice, and is shown in the graph on the right. **D.** RhoE expression reduces the induction of proliferation by TPA. Mice of the three genotypes (RhoE^+/+^, RhoE^+/*gt*^ and RhoE^*gt/gt*^, *n* = 3 of each one) were treated with a single dose of TPA (12.5 μg in 0.2 ml acetone) for 24 h. Percentage of PCNA positive nuclei determined by immunohistochemistry is plotted (**p < 0.01 and ***p < 0.001 in a 2 way ANOVA followed by Bonferroni's multiple comparisons test). **E.** TPA induces the expression of RhoE in skin. RhoE expression in skin samples from control and TPA-treated RhoE^+/+^ mice (as in A) was analyzed by Western blotting. Actin was used as a loading control.

To study whether RhoE could also behave as a tumor suppressor *in vivo*, we evaluated the susceptibility of RhoE hemizygous mice to chemically induced carcinogenesis, using a DMBA/TPA two-stage skin carcinogenesis protocol [34, 35]. We could not use RhoE*^gt/gt^* mice in this experiment because of their short lifespan [27]. Papillomas started to appear at the same time in RhoE*^+/gt^* and in RhoE*^+/+^* mice. However, the number of tumors per mice was significantly higher in the RhoE*^+/gt^* group than in wild type mice (Figure 4A). Moreover, tumors grew significantly faster in RhoE*^+/gt^* than in RhoE*^+/+^* mice, with 50% of them being larger than 2 mm at week 12 of treatment in the RhoE*^+/gt^* mice and at week 17 in the wild types (Figure 4B). Finally, we analyzed the progression to carcinomas after stopping the TPA treatment, finding that the conversion rate was double in RhoE*^+/gt^* than in RhoE*^+/+^* mice (Figure 4C). Therefore, a decrease in RhoE expression contributes to tumor progression.

Additionally, we analyzed the proliferative response in the skin after a single TPA dose administered topically. In this case, we used young animals (15 days old) and thus we were able to include also RhoE*^gt/gt^* mice. The number of proliferating cells, measured as PCNA positive nuclei, increased in the three groups 24 h after TPA treatment, compared to untreated controls (Figure 4D). However, this increase was significantly larger in RhoE*^+/gt^* and RhoE*^gt/gt^* mice than in RhoE*^+/+^* controls. Interestingly, Western blot analysis of skin lysates showed that the expression of RhoE in the skin of wild type animals increased after a single dose of TPA (Figure 4E).

### RhoE silencing increases the metastatic potential of MDA-MB-231 cells

**Figure 5:**
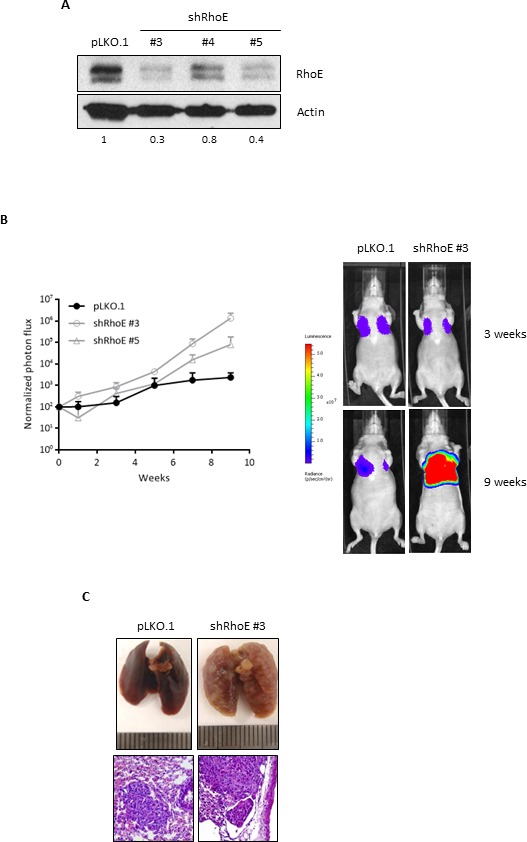
RhoE expression reduces metastatic potential of MDA-MB-231 cells **A.** MDA-MB-231-TGL cells were transduced with control lentivirus (pLKO.1) or three different shRNA constructs to knock-down RhoE expression. Knock-down efficiency was analyzed by Western blotting. Numbers show the relative expression level of RhoE after quantification by densitometry. **B.** After injection of MDA-MB-231-TGL cells (control and RhoE knock-down, using two different shRNAs) in the tail vein of 6 nude mice (2 per construct), lung tumors were analyzed every week by *in vivo* bioluminescence imaging and normalized photon flux was plotted (left graph). The image on the right shows representative results of mice injected with control (pLKO.1) and shRhoE #3 MDA-MB-231-TGL cells. **C.** Lung colonization by MDA-MB-231 cells. At the end of the experiment, lungs were removed and inspected to confirm the presence of tumors resulting from the injection of control (pLKO.1, left) and RhoE knocked-down (shRhoE #3, right) MDA-MB-231-TGL cells. Hematoxylin and eosin staining (bottom images, 200x) revealed no differences between control- and shRhoE- induced metastases tumors.

Finally, we wanted to test whether RhoE could be involved in metastasis. By using shRNA, we were able to achieve efficient RhoE expression knock-down (up to 68%) in breast carcinoma MDA-MB-231-TGL cells, which contain a GFP-luciferase cassette (Figure 5A). To test their metastatic potential, control and RhoE knock-down (shRhoE #3 and #5) MDA-MB-231-TGL cells were injected in the tail vein of nude mice and the appearance of metastases was followed by *in vivo* bioluminescence [36]. After 9 weeks, all the mice showed lung metastases, as expected, but mice injected with shRhoE cells showed significantly higher metastatic signals than vector-bearing controls (Figure 5B). After necropsy, visual inspection of the lungs confirmed the higher colonization by shRhoE cells compared to pLKO.1 control cells, although hematoxylin and eosin staining showed no obvious differences between control- and shRhoE- induced metastases (Figure 5C). From these experiments, we can conclude that a reduction in RhoE expression increases the metastatic potential of tumor cells *in vivo*, suggesting that RhoE is a suppressor of metastasis.

## DISCUSSION

In this work we have addressed the possible implication of RhoE in tumorigenesis. For this purpose, we have used primary MEFs and mice lacking RhoE expression (RhoE*^gt/gt^*), as well as shRNA to knock-down RhoE expression in cancer cells. Our results show that lack of RhoE expression suppresses contact inhibition, facilitates spontaneous immortalization and oncogenic transformation and increases tumorigenesis and metastatic potential of tumor cells.

The role of RhoE in cancer is currently unclear. Previous studies with different tumor types have suggested a positive correlation of RhoE expression with tumor malignancy [20-26] but also a tumor suppressive function for RhoE [7, 12-19]. Our *in vitro* and *in vivo* results clearly support that RhoE contributes to tumor suppression. The contrasting evidence regarding the role of RhoE in tumorigenesis could be due to differences in cell or tumor types, alterations in regulators or mediators of RhoE function or experimental details. Regarding cell type specificity, our data with primary fibroblasts and epidermal carcinogenesis is in agreement with evidence of a tumor suppressor role of RhoE in mesenchymal tumors [12] or squamous cell carcinoma [15]. Further examples of cell-type specificity are the reports of tumor suppressive function in liver tumors [13, 14] or oncogenic in lung tumors [20, 21]. However for other tumor types (gastric, prostate and colorectal carcinoma) both functions have been reported [7, 16, 17, 22-25]. Our results suggest that RhoE tumor suppressive function is mediated at least in part by a mechanism involving nuclear translocation of p27^Kip1^. Interestingly, p27^Kip1^ localization can be a marker for prognosis and response to treatment in several different types of cancer [38]. It would be interesting to correlate the different roles of RhoE in tumors with p27^Kip1^ levels and localization.

Although the expression of RhoE is dispensable for cell cycle progression in low density conditions, it is necessary for the correct control of cellular proliferation in high density cultures of primary MEFs. Contact inhibition is a mechanism to inhibit cell proliferation that is lost during tumorigenesis [39]. It controls cell number even in the presence of mitogens. Contact inhibited cells do not enter senescence and are viable after replating [40]. The best characterized event leading to contact inhibition is the induction of p27^Kip1^ expression, mediated by cadherins [28-30]. In fact, it has been shown that p27^Kip1^ induction, leading to contact inhibition, could be suppressing geroconversion which, in turn, is induced by mTOR mediated upregulation of the cell cycle inhibitor p21^Cip1^ [40]. Our results show that, p27^Kip1^ and RhoE are both accumulated in primary MEFs and breast carcinoma cells when they are contact-inhibited. These two proteins show the same expression kinetics along the cell cycle and are degraded through, at least, a similar mechanism involving the E3 Ubiquitin ligase Skp2 and the proteasome [9, 41, 42]. Besides, the expression of both proteins is downregulated by miR-200b in colorectal cancer [17]. All these data and our results presented here suggest that p27^Kip1^ and RhoE may have related functions in controlling excessive proliferation.

Primary fibroblasts lacking RhoE expression are able to reach higher densities in culture than wild type cells, despite the normal induction of p27^Kip1^. However, for p27^Kip1^ to behave as a cell cycle inhibitor, it needs to be translocated to the cell nucleus. Nuclear import of p27^Kip1^ depends on several phosphorylation events and interaction with different proteins [43]. In primary MEFs and MCF7 cells lacking RhoE expression, p27^Kip1^ is not properly translocated to the nucleus. This could explain why these cells do not seem to be contact inhibited. The mechanism by which RhoE contributes to the regulation of p27^Kip1^ localization remains to be determined. It has been recently shown that RhoE is necessary for proper nuclear translocation of the Notch intracellular domain (NICD) by forming a complex with importins in squamous cell carcinomas [15]. It is feasible that RhoE could use a similar mechanism involving formation of importin-p27^Kip1^ complexes to mediate p27^Kip1^ nuclear translocation.

In our study, we focused on two cellular and animal models to study the impact of RhoE in tumorigenesis. First, we show here that lack of RhoE expression increases the susceptibility of primary MEFs to oncogenic transformation by E1A/Ras^V12^, in agreement with a previous work reporting that RhoE overexpression inhibits Ras transformation of NIH3T3 fibroblasts [6]. Second, our results show the importance of RhoE *in vivo* in controlling chemically induced proliferation, tumor formation and progression in the skin. It has been reported that RhoE expression is upregulated in the skin by genotoxic stress [10, 11] and it can control proliferation and differentiation of keratinocytes *in vitro* by regulating Notch1 signaling [15, 44]. We now show that TPA treatment results in the accumulation of RhoE in the skin. This observation could be related to the reported phosphorylation of RhoE by PKC that could lead to stabilization of RhoE [45, 46]. The lack of RhoE expression also results in increased proliferation in the skin after TPA treatment, indicating that RhoE contributes to controlling excessive proliferation in this context. Furthermore, we show that RhoE deficiency promotes both initiation (incidence of papillomas) and progression (conversion to carcinomas) in a skin chemical carcinogenesis model. In addition, decreased RhoE expression also increases the metastatic potential of tumor cells *in vivo*, suggesting that RhoE is a suppressor of metastasis, at least of the last stages of the process related with infiltration and colonization [37]. ROCK activity is necessary for the ameboid movement that allows migration and invasion of cancer cells [19, 47]. As RhoE inhibits ROCK activity, this could be a mechanism by which it may contribute to negatively regulate tumor metastasis.

In summary, our results indicate that RhoE is involved in the control of contact inhibition by regulating p27^Kip1^ localization, negatively regulates excessive proliferation induced by oncogenes and carcinogens and limits metastatic potential of cancer cells, and therefore suggest an important role of RhoE in tumor suppression.

## MATERIALS AND METHODS

### Animal procedures

All animal procedures were approved by the local ethics committee (Ethics Committee for Animal Welfare of the Universidad CEU Cardenal Herrera, ID#CEBA-09/006), met the local guidelines (Spanish law 53/2013), European regulations (EU directive 86/609) and Standards for Use of Laboratory Animals A5388-01 (NIH). All efforts were made to minimize the number of animals used and their suffering. Mice were sacrificed by cervical dislocation.

Mice deficient for RhoE expression (RhoE*^gt/gt^*) were generated by insertion of a gene-trap cassette in intron 2 of the gene [48]. The resulting phenotype has been described previously [27].

Hsd:Athymic Nude-*Foxn1^nu^* immunocompromised mice were obtained from Harlan Laboratories (Indianapolis, IN, USA).

### Cell culture and proliferation analysis

All cells were maintained in high glucose Dulbecco's modified Eagle's (DMEM) supplemented with 10% FBS.

Primary Mouse Embryonic Fibroblasts (MEFs) were obtained as described [49] and used at early passage (P2-P5). The human breast adenocarcinoma-derived MCF7 cell line was obtained from the European Collection of Cell Cultures (ECACC). Breast cancer-derived MDA-MB-231 cells infected with a triple-fusion protein reporter construct encoding herpes simplex virus thymidine kinase 1, green fluorescent protein (GFP) and firefly luciferase (TGL) for bioluminescent tracking (MDA-MB-231-TGL cells) were kindly provided by Dr. J. Massagué [36].

For proliferation assays, primary MEFs were plated in triplicates at 2×10^4^ cells on 35 mm dishes. Every 48 h, starting 24 h after plating (time 0), cells were fixed in 4% formaldehyde (FA), and stained with 1% crystal violet for 30 min. After solubilization in 15% acetic acid, absorbance was measured at 570 nm.

For high density culture assays, primary MEFs were plated at 4×10^4^ cells on 35 mm dishes and the culture medium was changed every 48 h. 15 days after plating, cells were fixed, stained and cell density was measured as described above.

For cell cycle analysis, primary MEFs were plated at 7×10^5^ cells on 100 mm dishes and maintained in DMEM with 10% FBS for 48 h (Asynchronous), without FBS for further 48 h (No serum) or FBS was added for 16 h after 48 h serum starvation (Re-stimulated). Cells were collected, fixed, stained with propidium iodide and analyzed by flow cytometry as previously described [42, 50].

### *In vitro* transformation assays

For immortalization assays (3T3 protocol), 1.75×10^5^ MEFs of each genotype were plated on 35 mm dishes. Every three days cells were trypsinized, counted and replated at the same density. The number of divisions (population doubling level, PDL) was calculated using the following formula: PDL = 3.32 × (logNf-logNi), where Ni is the initial number of cells and Nf the number of cells collected for each point [51]. Cells were considered senescent when no significant increase in cell number was observed for three consecutive passages and immortalized when cell number increased for three consecutive passages after senescence.

For oncogene transformation assays, RhoE*^+/+^* and RhoE*^gt/gt^* MEFs were infected with empty pLPC vector (control), pLPC-Ras^V12^ or pLPC-E1A/Ras^V12^ and seeded at a density of 2×10^3^ cells on 100 mm dishes per triplicate. Cells were grown in DMEM with 10% FBS for 1-3 weeks and then they were fixed with formaldehyde 4% and stained with crystal violet for colony visualization.

### *In vivo* tumorigenesis assays

RhoE*^+/+^* and RhoE*^gt/gt^* MEFs previously infected with pLPC-E1A/Ras^V12^ were subcutaneously injected into both posterior flanks of 16 nude mice, respectively (4×10^5^ cells in 100 μl of PBS per injection). Tumor volume was measured with a caliper and calculated every four days by using the following formula: V=AxB^2^/2 (cm^3^), where A is the major diameter and B the perpendicular tumor diameter. After 5 weeks, mice were sacrificed and tumors were removed and processed for histological analysis.

For the chemical induction of papillomas *in vivo*, we used the DMBA-TPA two-step carcinogenesis protocol, in which DMBA causes a mutation in Ha-Ras as the initiating event and the tumor promoter TPA activates PKC [34, 35]. A single dose of DMBA (32 μg in 0.2 ml acetone) followed by weekly doses of TPA (12.5 μg in 0.2 ml acetone) for 15 weeks were applied to the shaved back of 14 RhoE*^+/+^* and 14 RhoE*^+/gt^* mice. Lesions were counted weekly for 40 weeks. At the end of the experiment, mice were sacrificed and tumors were processed for histopathology analysis and classified as epithelial hyperplasia, papilloma or squamous cell carcinoma.

### Western blotting and immunohistochemistry

Cell samples were processed for Western blotting and for subcellular fractionation as previously described [8, 42, 52].

The following antibodies were used for Western blotting: anti-RhoE (Merck Millipore, Darmstadt, Germany); anti-p27^Kip1^ and anti-p21^Cip1^ (Santa Cruz Biotechnology, Santa Cruz, CA, USA); anti-Actin and anti-β-tubulin (Sigma-Aldrich, St. Louis, MO, USA); anti-lamin A (Cell Signaling, Beverly, MA, USA). Blots were developed using enhanced chemiluminescence (ECL Plus, GE Healthcare Life Sciences, Fairfield, CT, USA).

For immunohistochemistry, PCNA antibodies (Santa Cruz Biotechnology) were used on paraffin embedded skin sections.

### RhoE silencing

Control lentivirus (pLKO.1) or three different RhoE shRNA constructs from Mission Library (Sigma-Aldrich) were used to knock-down RhoE expression. The shRNA sequences were:

shRhoE #3 (TRCN330303):CCGGATCCTAATCAGAACGTGAAATCTCGAGATTTCACGTTCTGATTAGGATTTTTTG
shRhoE #4 (TRCN330304):CCGGCGGACAGATGTTAGTACATTACTCGAGTAATGTACTAACATCTGTC CGTTTTTG
shRhoE #5 (TRCN330305):CCGGGAGAGCCACAAAGCGGATTTCCTCGAGGAAATCCGCTTTGTGGCTCTCTTTTTG

We used shRhoE #3, shRhoE #4 and shRhoE #5 to transduce MDA-MB-231-TGL cells and shRhoE #3 to transduce MCF7 cells. After infection, cells were selected with 2.5 μg/ml Puromycin.

### Experimental metastasis assay and bioluminescence imaging

Control and two RhoE knocked-down (shRhoE #3 and shRhoE #5) MDA-MB-231-TGL cells from subconfluent cultures were injected (1×10^6^ in 0.1 ml PBS) into the tail vein of nude mice. For *in vivo* bioluminescence imaging, mice were anesthetized and injected intraperitoneally with 3 mg of d-luciferin (15 mg/ml in PBS). Imaging was completed between 5 and 30 min after injection with a Xenogen IVIS (IVISR Lumina II) system coupled to Living Image acquisition and analysis software (Xenogen Corporation). For bioluminescence intensity (BLI) plots, photon flux was calculated as described [36]. Measurements were performed once a week starting 1 week after tail vein injection and up to 9 weeks.

### Statistical analysis

Data were analyzed by the Student's *t* test, ANOVA or the Mantel-Cox test using the GraphPad Prism software. Differences were considered significant if *p* < 0.05.

## References

[1] Guasch RM, Scambler P, Jones GE, Ridley AJ (1998). RhoE regulates actin cytoskeleton organization and cell migration. Mol Cell Biol.

[2] Komander D, Garg R, Wan PT, Ridley AJ, Barford D (2008). Mechanism of multi-site phosphorylation from a ROCK-I:RhoE complex structure. The EMBO journal.

[3] Riento K, Guasch RM, Garg R, Jin B, Ridley AJ (2003). RhoE binds to ROCK I and inhibits downstream signaling. Mol Cell Biol.

[4] Foster R, Hu KQ, Lu Y, Nolan KM, Thissen J, Settleman J (1996). Identification of a novel human Rho protein with unusual properties: GTPase deficiency and *in vivo* farnesylation. Mol Cell Biol.

[5] Chardin P (2006). Function and regulation of Rnd proteins. Nat Rev Mol Cell Biol.

[6] Villalonga P, Guasch RM, Riento K, Ridley AJ (2004). RhoE inhibits cell cycle progression and Ras-induced transformation. Mol Cell Biol.

[7] Bektic J, Pfeil K, Berger AP, Ramoner R, Pelzer A, Schafer G, Kofler K, Bartsch G, Klocker H (2005). Small G-protein RhoE is underexpressed in prostate cancer and induces cell cycle arrest and apoptosis. The Prostate.

[8] Poch E, Minambres R, Mocholi E, Ivorra C, Perez-Arago A, Guerri C, Perez-Roger I, Guasch RM (2007). RhoE interferes with Rb inactivation and regulates the proliferation and survival of the U87 human glioblastoma cell line. Exp Cell Res.

[9] Lonjedo M, Poch E, Mocholi E, Hernandez-Sanchez M, Ivorra C, Franke TF, Guasch RM, Perez-Roger I (2013). The Rho family member RhoE interacts with Skp2 and is degraded at the proteasome during cell cycle progression. The Journal of biological chemistry.

[10] Boswell SA, Ongusaha PP, Nghiem P, Lee SW (2007). The protective role of a small GTPase RhoE against UVB-induced DNA damage in keratinocytes. The Journal of biological chemistry.

[11] Ongusaha PP, Kim HG, Boswell SA, Ridley AJ, Der CJ, Dotto GP, Kim YB, Aaronson SA, Lee SW (2006). RhoE is a pro-survival p53 target gene that inhibits ROCK I-mediated apoptosis in response to genotoxic stress. Current biology : CB.

[12] Belgiovine C, Frapolli R, Bonezzi K, Chiodi I, Favero F, Mello-Grand M, Dei Tos AP, Giulotto E, Taraboletti G, D'Incalci M, Mondello C (2010). Reduced expression of the ROCK inhibitor Rnd3 is associated with increased invasiveness and metastatic potential in mesenchymal tumor cells. PloS one.

[13] Grise F, Sena S, Bidaud-Meynard A, Baud J, Hiriart JB, Makki K, Dugot-Senant N, Staedel C, Bioulac-Sage P, Zucman-Rossi J, Rosenbaum J, Moreau V (2012). Rnd3/RhoE Is down-regulated in hepatocellular carcinoma and controls cellular invasion. Hepatology.

[14] Luo H, Dong Z, Zou J, Zeng Q, Wu D, Liu L (2012). Down-regulation of RhoE is associated with progression and poor prognosis in hepatocellular carcinoma. J Surg Oncol.

[15] Zhu Z, Todorova K, Lee KK, Wang J, Kwon E, Kehayov I, Kim HG, Kolev V, Dotto GP, Lee SW, Mandinova A (2014). Small GTPase RhoE/Rnd3 Is a Critical Regulator of Notch1 Signaling. Cancer Res.

[16] Chen J, Zhou H, Li Q, Qiu M, Li Z, Tang Q, Liu M, Zhu Y, Huang J, Lang N, Liu Z, Deng Y, Zhang S, Bi F (2011). Epigenetic modification of RhoE expression in gastric cancer cells. Oncol Rep.

[17] Fu Y, Liu X, Zhou N, Du L, Sun Y, Zhang X, Ge Y (2014). MicroRNA-200b stimulates tumour growth in TGFBR2-null colorectal cancers by negatively regulating p27/kip1. J Cell Physiol.

[18] Hidalgo-Carcedo C, Hooper S, Chaudhry SI, Williamson P, Harrington K, Leitinger B, Sahai E (2011). Collective cell migration requires suppression of actomyosin at cell-cell contacts mediated by DDR1 and the cell polarity regulators Par3 and Par6. Nature cell biology.

[19] Pinner S, Sahai E (2008). PDK1 regulates cancer cell motility by antagonising inhibition of ROCK1 by RhoE. Nature cell biology.

[20] Cuiyan Z, Jie H, Fang Z, Kezhi Z, Junting W, Susheng S, Xiaoli F, Ning L, Xinhua M, Zhaoli C, Kang S, Bin Q, Baozhong L, Sheng C, Meihua X (2007). Overexpression of RhoE in Non-small Cell Lung Cancer (NSCLC) is associated with smoking and correlates with DNA copy number changes. Cancer biology & therapy.

[21] Zhang C, Zhou F, Li N, Shi S, Feng X, Chen Z, Hang J, Qiu B, Li B, Chang S, Wan J, Shao K, Xing X, Tan X, Wang Z, Xiong M (2007). Overexpression of RhoE has a prognostic value in non-small cell lung cancer. Ann Surg Oncol.

[22] Feng B, Li K, Zhong H, Ren G, Wang H, Shang Y, Bai M, Liang J, Wang X, Fan D (2013). RhoE promotes metastasis in gastric cancer through a mechanism dependent on enhanced expression of CXCR4. PloS one.

[23] Trojan L, Schaaf A, Steidler A, Haak M, Thalmann G, Knoll T, Gretz N, Alken P, Michel MS (2005). Identification of metastasis-associated genes in prostate cancer by genetic profiling of human prostate cancer cell lines. Anticancer research.

[24] Zhou J, Li K, Gu Y, Feng B, Ren G, Zhang L, Wang Y, Nie Y, Fan D (2011). Transcriptional up-regulation of RhoE by hypoxia-inducible factor (HIF)-1 promotes epithelial to mesenchymal transition of gastric cancer cells during hypoxia. Biochemical and biophysical research communications.

[25] Zhou J, Yang J, Li K, Mo P, Feng B, Wang X, Nie Y, Fan D (2013). RhoE is associated with relapse and prognosis of patients with colorectal cancer. Ann Surg Oncol.

[26] Klein RM, Aplin AE (2009). Rnd3 regulation of the actin cytoskeleton promotes melanoma migration and invasive outgrowth in three dimensions. Cancer Res.

[27] Mocholi E, Ballester-Lurbe B, Arque G, Poch E, Peris B, Guerri C, Dierssen M, Guasch RM, Terrado J, Perez-Roger I (2011). RhoE deficiency produces postnatal lethality, profound motor deficits and neurodevelopmental delay in mice. PloS one.

[28] St Croix B, Sheehan C, Rak JW, Florenes VA, Slingerland JM, Kerbel RS (1998). E-Cadherin-dependent growth suppression is mediated by the cyclin-dependent kinase inhibitor p27(KIP1). The Journal of cell biology.

[29] Levenberg S, Yarden A, Kam Z, Geiger B (1999). p27 is involved in N-cadherin-mediated contact inhibition of cell growth and S-phase entry. Oncogene.

[30] Motti ML, Califano D, Baldassarre G, Celetti A, Merolla F, Forzati F, Napolitano M, Tavernise B, Fusco A, Viglietto G (2005). Reduced E-cadherin expression contributes to the loss of p27kip1-mediated mechanism of contact inhibition in thyroid anaplastic carcinomas. Carcinogenesis.

[31] Blagosklonny MV (2002). Are p27 and p21 cytoplasmic oncoproteins?. Cell cycle.

[32] Serres MP, Zlotek-Zlotkiewicz E, Concha C, Gurian-West M, Daburon V, Roberts JM, Besson A (2011). Cytoplasmic p27 is oncogenic and cooperates with Ras both *in vivo* and *in vitro*. Oncogene.

[33] Todaro GJ, Green H (1963). Quantitative studies of the growth of mouse embryo cells in culture and their development into established lines. The Journal of cell biology.

[34] Kemp CJ (2005). Multistep skin cancer in mice as a model to study the evolution of cancer cells. Seminars in cancer biology.

[35] Quintanilla M, Brown K, Ramsden M, Balmain A (1986). Carcinogen-specific mutation and amplification of Ha-ras during mouse skin carcinogenesis. Nature.

[36] Minn AJ, Gupta GP, Siegel PM, Bos PD, Shu W, Giri DD, Viale A, Olshen AB, Gerald WL, Massague J (2005). Genes that mediate breast cancer metastasis to lung. Nature.

[37] Nguyen DX, Bos PD, Massague J (2009). Metastasis: from dissemination to organ-specific colonization. Nat Rev Cancer.

[38] Chu IM, Hengst L, Slingerland JM (2008). The Cdk inhibitor p27 in human cancer: prognostic potential and relevance to anticancer therapy. Nat Rev Cancer.

[39] Hanahan D, Weinberg RA (2011). Hallmarks of cancer: the next generation. Cell.

[40] Leontieva OV, Demidenko ZN, Blagosklonny MV (2014). Contact inhibition and high cell density deactivate the mammalian target of rapamycin pathway, thus suppressing the senescence program. Proceedings of the National Academy of Sciences of the United States of America.

[41] Lu Z, Hunter T (2010). Ubiquitylation and proteasomal degradation of the p21(Cip1), p27(Kip1) and p57(Kip2) CDK inhibitors. Cell cycle.

[42] Andreu EJ, Lledo E, Poch E, Ivorra C, Albero MP, Martinez-Climent JA, Montiel-Duarte C, Rifon J, Perez-Calvo J, Arbona C, Prosper F, Perez-Roger I (2005). BCR-ABL induces the expression of Skp2 through the PI3K pathway to promote p27Kip1 degradation and proliferation of chronic myelogenous leukemia cells. Cancer Res.

[43] Borriello A, Cucciolla V, Oliva A, Zappia V, Della Ragione F (2007). p27Kip1 metabolism: a fascinating labyrinth. Cell cycle.

[44] Liebig T, Erasmus J, Kalaji R, Davies D, Loirand G, Ridley A, Braga VM (2009). RhoE Is required for keratinocyte differentiation and stratification. Molecular biology of the cell.

[45] Madigan JP, Bodemann BO, Brady DC, Dewar BJ, Keller PJ, Leitges M, Philips MR, Ridley AJ, Der CJ, Cox AD (2009). Regulation of Rnd3 localization and function by protein kinase C alpha-mediated phosphorylation. Biochem J.

[46] Riento K, Totty N, Villalonga P, Garg R, Guasch R, Ridley AJ (2005). RhoE function is regulated by ROCK I-mediated phosphorylation. The EMBO journal.

[47] Pinner S, Sahai E (2008). Imaging amoeboid cancer cell motility *in vivo*. J Microsc.

[48] Zambrowicz BP, Friedrich GA, Buxton EC, Lilleberg SL, Person C, Sands AT (1998). Disruption and sequence identification of 2,000 genes in mouse embryonic stem cells. Nature.

[49] Perez-Roger I, Kim SH, Griffiths B, Sewing A, Land H (1999). Cyclins D1 and D2 mediate myc-induced proliferation via sequestration of p27(Kip1) and p21(Cip1). The EMBO journal.

[50] Albero MP, Vaquer JM, Andreu EJ, Villanueva JJ, Franch L, Ivorra C, Poch E, Agirre X, Prosper F, Perez-Roger I (2010). Bortezomib decreases Rb phosphorylation and induces caspase-dependent apoptosis in Imatinib-sensitive and -resistant Bcr-Abl1-expressing cells. Oncogene.

[51] Hayflick L (1973). Tissue culture: methods and applications.

[52] Ishida N, Hara T, Kamura T, Yoshida M, Nakayama K, Nakayama KI (2002). Phosphorylation of p27Kip1 on serine 10 is required for its binding to CRM1 and nuclear export. The Journal of biological chemistry.

